# Secondary forest regeneration benefits old-growth specialist bats in a fragmented tropical landscape

**DOI:** 10.1038/s41598-018-21999-2

**Published:** 2018-02-28

**Authors:** Ricardo Rocha, Otso Ovaskainen, Adrià López-Baucells, Fábio Z. Farneda, Erica M. Sampaio, Paulo E. D. Bobrowiec, Mar Cabeza, Jorge M. Palmeirim, Christoph F. J. Meyer

**Affiliations:** 1 Centre for Ecology, Evolution and Environmental Changes – cE3c, Faculty of Sciences, University of Lisbon, 1749–016 Lisbon, Portugal; 2 Biological Dynamics of Forest Fragments Project, National Institute for Amazonian Research and Smithsonian Tropical Research Institute, 69011–970 Manaus, Brazil; 3 Metapopulation Research Centre, Faculty of Biosciences, University of Helsinki, FI-00014 Helsinki, Finland; 4 Centre for Biodiversity Dynamics, Department of Biology, Norwegian University of Science and Technology, N-7491 Trondheim, Norway; 5 Granollers Museum of Natural Sciences, 08402 Catalunya, Spain; 6 Department of Ecology/PPGE, Federal University of Rio de Janeiro, 21941–901 Rio de Janeiro, Brazil; 7 University of Ulm, Institute of Evolutionary Ecology and Conservation Genomics, 89069 Ulm, Germany; 8 Ecosystems and Environment Research Centre (EERC), School of Environment and Life Sciences, University of Salford, Salford, M5 4WT United Kingdom

## Abstract

Tropical forest loss and fragmentation are due to increase in coming decades. Understanding how matrix dynamics, especially secondary forest regrowth, can lessen fragmentation impacts is key to understanding species persistence in modified landscapes. Here, we use a whole-ecosystem fragmentation experiment to investigate how bat assemblages are influenced by the regeneration of the secondary forest matrix. We surveyed bats in continuous forest, forest fragments and secondary forest matrix habitats, ~15 and ~30 years after forest clearance, to investigate temporal changes in the occupancy and abundance of old-growth specialist and habitat generalist species. The regeneration of the second growth matrix had overall positive effects on the occupancy and abundance of specialists across all sampled habitats. Conversely, effects on generalist species were negligible for forest fragments and negative for secondary forest. Our results show that the conservation potential of secondary forests for reverting faunal declines in fragmented tropical landscapes increases with secondary forest age and that old-growth specialists, which are often of most conservation concern, are the greatest beneficiaries of secondary forest maturation. Our findings emphasize that the transposition of patterns of biodiversity persistence in island ecosystems to fragmented terrestrial settings can be hampered by the dynamic nature of human-dominated landscapes.

## Introduction

Humanity’s global footprint is so ubiquitous and far-reaching that many argue that we now live in a new geological epoch, the Anthropocene^1^. Habitat loss and fragmentation are pervasive and conspicuous features of this new historical context, which, in combination with other human-related threats, are compelling the planet into a “sixth wave of extinction”^2,3^.

The scars of the Anthropocene defaunation are being carved deep into the planet’s biodiversity strongholds, the tropical forests^4^. As large swaths of old-growth forest give way to expanding humanized landscapes, species persisting in forest remnants are left to endure the pervasive consequences of increased isolation and decreased area^5^. Landscape-wide assemblage dynamics in fragments created in the aftermath of deforestation are dependent, to a large extent, on the nature of the matrix within which forest patches are embedded^6^. Conservation science has traditionally conceived the modified matrix as a “sea” of hostile habitat, in which fragments act as “islands” and this analogy has guided much of the theory and practice of the field^6,7^. However, equating forest fragments with island ecosystems, while appropriate in some situations, fails to accommodate the heterogeneous and dynamic nature of most present-day modified landscapes^8,9^.

Vertebrate assemblage dynamics in tropical land-bridge islands have painted a dire portrait of the consequences of forest fragmentation in true island systems^10–12^. Mainland studies that also construed fragments as true islands, have arrived at similar pessimistic narratives^13,14^. However, direct comparisons between these two systems (true islands vs mainland) have revealed that assemblages persisting in forest patches embedded in terrestrial human-dominated landscapes defy the patterns exhibited by their water-embedded analogues^9,15^.

Second growth nowadays constitutes the predominant type of forest cover across the tropics^16^, providing myriad services and natural products to human populations worldwide, and key habitat for countless forest-dwelling species^17,18^. Although some fragmentation-related extinctions can be averted by forest regeneration^14,15,19^, the role of second growth in biodiversity conservation remains controversial^20–22^. Central to the debate is the capacity of secondary forest to preserve old-growth specialist species and to buffer the impacts of fragmentation on assemblages living in forest remnants^16,17,23^.

**Figure 1:**
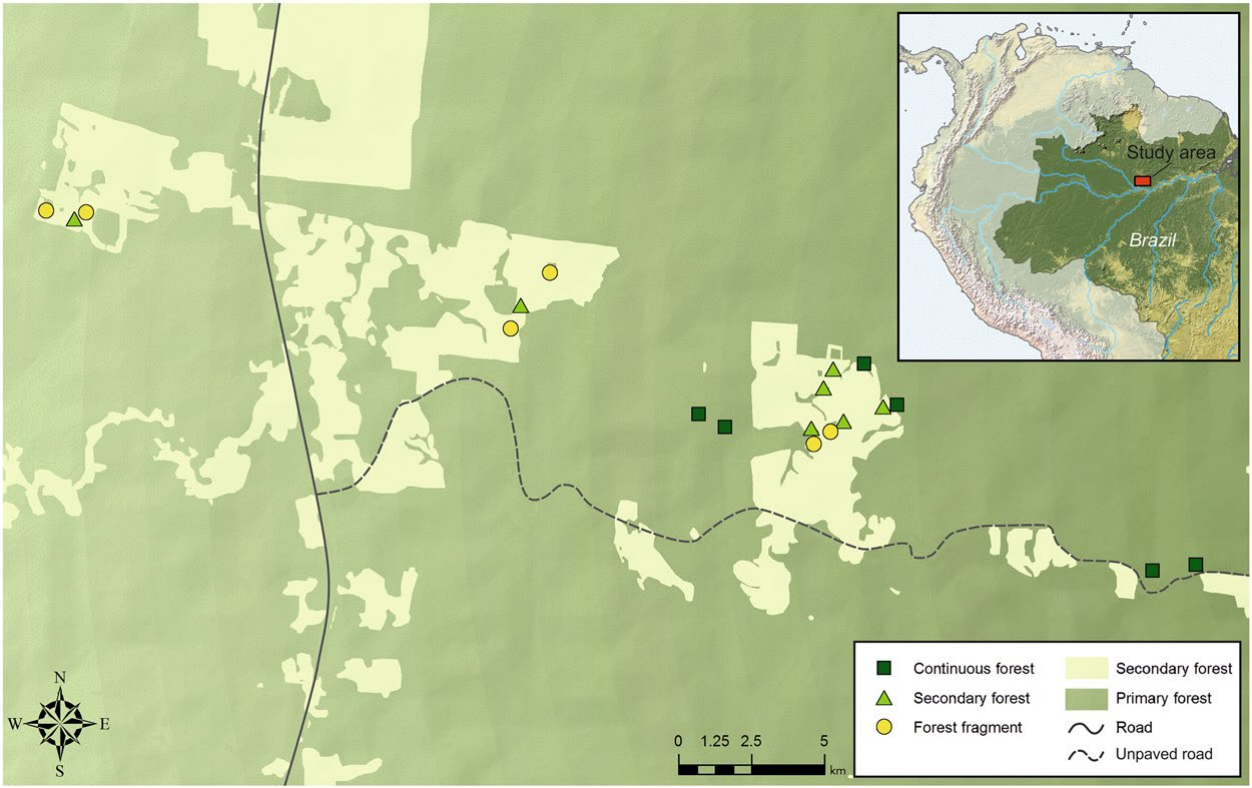
The Biological Dynamics of Forest Fragments Project (BDFFP), Central Amazon, Brazil. Light green represents secondary forest matrix and dark green continuous forest and forest fragments. Location of the study area within Brazil is shown in the map inset. The map was based on shapefiles provided by the BDFFP management team (http://pdbff.inpa.gov.br/) and was produced in ArcMap 10.3 (http://desktop.arcgis.com/en/arcmap/).

We surveyed bats, a taxon demonstrably sensitive to habitat modification^24^, in forest fragments and secondary forest sites, ~15 and ~30 years after forest clearance in the early 1980s at the Biological Dynamics of Forest Fragments Project (BDFFP), the world’s largest and longest-running fragmentation experiment, located in the central Brazilian Amazon^25^ (Fig. 1). Determining the responses of tropical species to habitat change is often hindered by the rarity of old-growth specialists for which data are often too sparse for reliable inference at the species level. This commonly leads to the exclusion of species captured less frequently (which are often of conservation concern) from the analysis or to several species being lumped together according to group membership (e.g. feeding guilds), thus preventing the detection of species-specific responses. Here, we overcome this difficulty by employing a joint species distribution modelling framework that combines species-specific models into a single hierarchical model that allows the detection of the relationship between environmental variables and species responses simultaneously at the species and community levels^26,27^. Our aim was to examine the effect of matrix regeneration between ~1996 and ~2011 on old-growth specialist and habitat generalist phyllostomid bat species (and *Pteronotus parnellii*) across the three main habitats of the BDFFP: continuous primary forest, primary forest fragments and secondary forest matrix. We predicted that the maturation between study periods of the secondary forest surrounding forest fragments would provide extra resources for old-growth specialists, leading to increases in occupancy and abundance in this group both within fragments and the secondary regrowth matrix. Conversely, we expected that the successional advance of the secondary vegetation would have diminished the availability of food resources for generalist bats (many of which feed on early-successional plants), hence reducing their abundance in the same habitats. Additionally, since similarity in structure and floristic composition between secondary and primary forests increases with regeneration time^16,28^ we predicted bat assemblage similarity between continuous forest and secondary forest to be higher ~30 years after forest clearance (~2011) than half-way through the study period (~15 years after forest clearance; ~1996). Similarly, due to a reduction in fragment-matrix contrast, we predicted that assemblage similarity between forest fragments and continuous forest was going to increase over the same period.

## Results

We captured 4,028 bats in the first period (35, 33 and 22 species in continuous forest, forest fragments and secondary forest respectively; 20 species shared between the three habitats) and 2,081 bats in the second period (33, 34 and 35 species in continuous forest, forest fragments and secondary forest respectively; 26 species shared between the three habitats). Twenty-seven species were classified as specialists whereas 23 were classified as habitat generalists (Supplementary Table S1).

**Figure 2:**
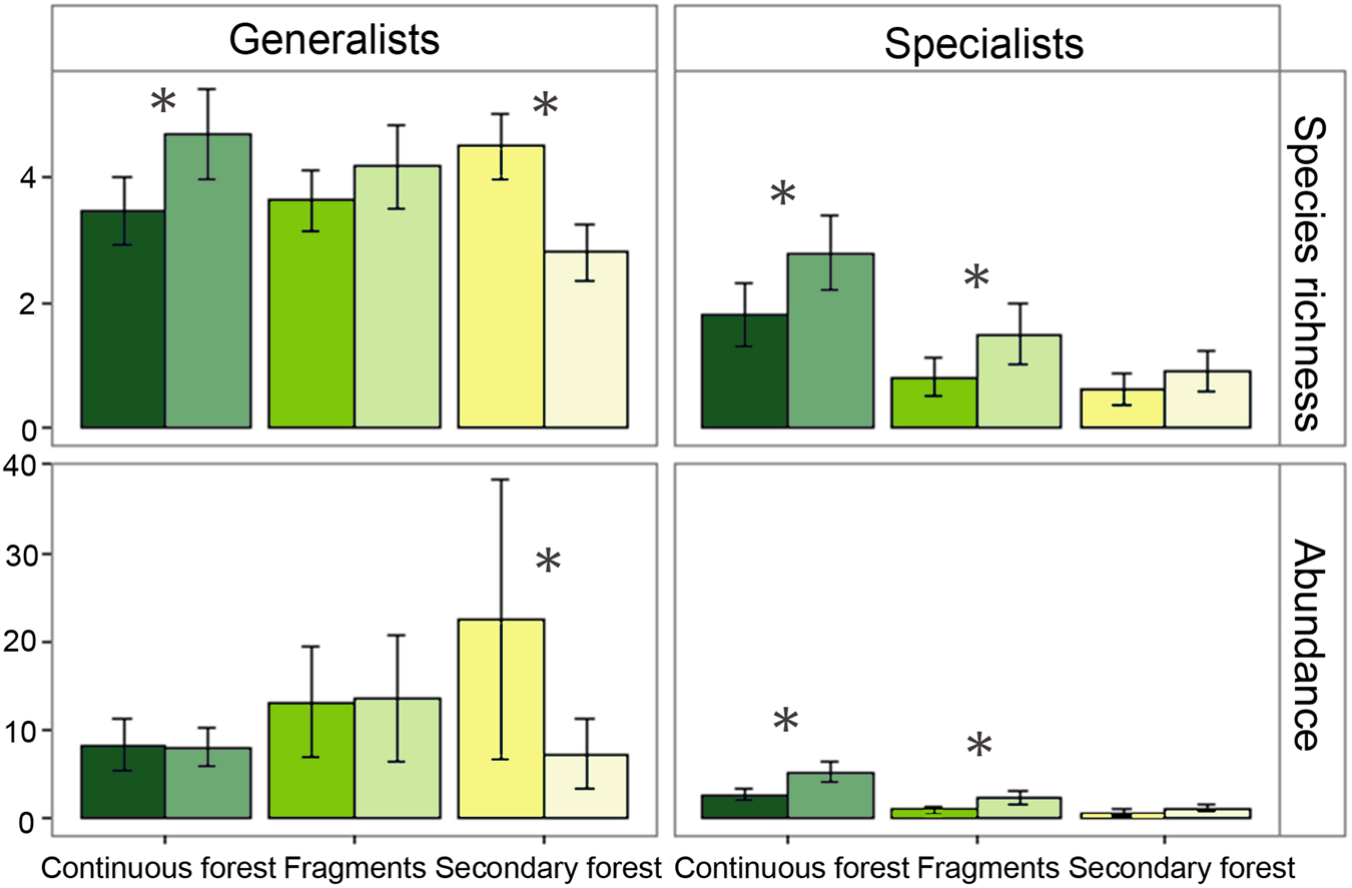
Bat species richness and abundance of generalist and specialist bats in continuous forest, fragments and secondary forest, ~15 years (dark-coloured bars) and ~30 years (lighter-coloured bars) after experimental forest clearance. Plotted are the predictions of the mean number of species and the mean number of individuals ( ± posterior standard deviation) captured per survey visit. Capture effort was standardized within each habitat category and thus the results are comparable only between periods but not across habitat types. *Asterisks* stand for high statistical support (posterior probability > 95%) for the predictions being higher or lower ~30 years after experimental forest clearance (2011–2013) than ~15 years after experimental forest clearance (1996–2002). Species’ habitat affinities are reported in Table S1 (for classification description see Methods) and results for all species combined are provided in Supplementary Fig. S1.

Our modelling results revealed that the regeneration of the matrix between the two periods had overall positive effects on the estimated occupancy and abundance of specialist bats in secondary forest sites and fragments, whereas effects on generalist species were negligible (fragments) or negative (secondary forest) (Fig. 2). Model predictions indicate that for specialist bats the mean number of species expected to be captured during a survey visit nearly doubled in fragments (0.81 in ~1996; 1.5 in ~2011) while remaining virtually unchanged for generalist species (3.63 in ~1996; 4.17 in ~2011). In secondary forest, this figure also increased for specialist bats (0.62 in ~1996; 0.91 in ~2011), while decreasing for generalist species (4.5 in ~1996; 2.81 in ~2011) and in continuous forest increased for both groups (1.81 in ~1996; 2.79 in ~2011 (specialists) and 3.47 in ~1996; 4.7 in ~2011 (generalists)) (Fig. 2). The mean number of individuals captured during a given survey varied little between the first and second period in continuous forest and fragments but decreased by nearly 2/3 in secondary forest (from 23.24 in ~1996 to 8.39 in ~2011) (Supplementary Fig. S2). In this habitat, generalists and specialists exhibited opposite trends between periods, with the mean number of individuals of generalist species declining from 22.55 in ~1996 to 7.3 in ~2011 and the mean number of individuals of specialist species increasing from 0.68 to 1.1 in the same period (Fig. 2).

**Figure 3:**
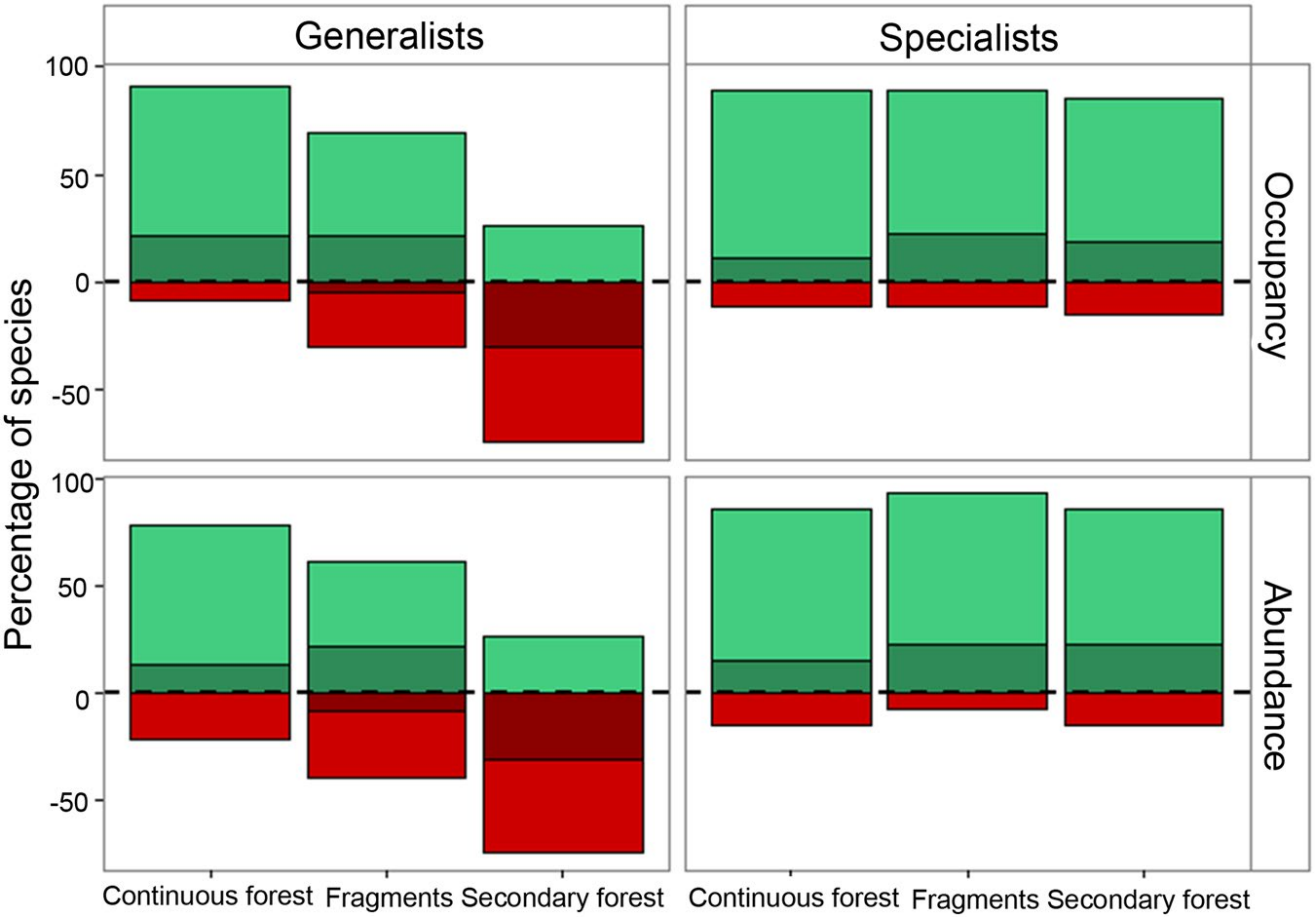
Change in species occupancy probability and abundance of generalist and specialist bats in continuous forest, fragments and secondary forest, ~15 years and ~30 years after experimental forest clearance. Plotted is the percentage of species with positive (green) and negative (red) changes in probability of occurrence and mean number of individuals predicted to be captured per survey visit between the first and second period (~15 and ~30 after experimental forest clearance). Dark and light colours represent respectively, percentage of species with high (posterior probability > 95%) and low statistical support (posterior probability < 95%). Predictions account for within-habitat differences in capture effort between the two periods. Species-specific values are reported in Supplementary Table S2; species’ habitat affinities are given in Supplementary Table S1 (for classification description see Methods).

Between ~1996 and ~2011, only 3 and 4 of the 27 species classified as specialists decreased in occupancy respectively in fragments and secondary forest. Furthermore, statistical support for these declines was limited (Fig. 3; Supplementary Table S2). During the same period, out of the 27 specialists, the abundance increased for 24 in fragments and for 23 in secondary forest. In contrast, of the 23 species classified as generalists, 7 declined in occupancy in fragments and 17 in secondary forest (high statistical support for 1 and 7 species, respectively) (Fig. 3; Supplementary Table S2). Seven generalist species declined in abundance in fragments and 17 in secondary forest (Fig. 3).

Assemblage similarity between continuous forest and fragments increased slightly with time for generalists when considering both occupancy and abundance but declined for specialists. For secondary forests, occupancy- and abundance-based assemblage similarities relative to continuous forest declined for both groups. However, statistical support for these trends was limited (Table 1; See Supplementary Table S3 for assemblage similarity comparisons for all species combined).

**Table 1:** Assemblage similarity between continuous forest and modified habitats (fragments and secondary forest), ~15 years and ~30 years after experimental forest clearance. We defined the similarity between two assemblages as the correlation between model-predicted occurrence probabilities or abundances (both log-transformed). The values in the table show posterior mean similarities between assemblages inhabiting continuous forests and modified habitats for the two study periods, as well as the posterior probability by which the similarities were lower in the first period than in the second.

Generalists	Fragments		Secondary Forest	
	*Occupancy*	*Abundance*	*Occupancy*	*Abundance*
*1996–2002*	0.64	0.71	0.60	0.68
*2011–2013*	0.76	0.79	0.51	0.56
*Posterior probability*	0.8	0.74	0.21	0.31
**Specialists**				
*1996–2002*	0.84	0.85	0.78	0.78
*2011–2013*	0.74	0.76	0.61	0.63
*Posterior probability*	0.16	0.16	0.09	0.09

## Discussion

There is a lack of studies that directly investigate temporal trends in wildlife responses to fragmentation and even fewer that evaluate how matrix use changes through time (but see^5,29^). Consequently, in contrast to the evaluation of species responses to spatial features, which has received some attention in the bat conservation literature, temporal variation, and in particular how bat responses to fragmentation are shaped by changes in matrix condition, remains little explored^24^. Here, we show that most phyllostomid bat species and *Pteronotus parnellii* benefited from the increased permeability of the matrix associated with the maturation of the secondary regrowth surrounding the BDFFP fragments, and that specialist and generalist species exhibited contrasting responses to matrix regeneration across the study landscape.

As hypothesized, we found that the maturation of second growth surrounding the BDFFP fragments lead to a landscape-wide increase in the occupancy and abundance of specialists, while reducing the occupancy and abundance of generalists in secondary forest sites. Our results therefore mirror the recovery documented for beetle^30^ and bird^31^ assemblages following the development of secondary vegetation in the matrix at the BDFFP. However, it is worth emphasizing that the BDFFP is surrounded by vast expanses of continuous forest harbouring healthy source populations and is buffered from selective logging, fires, species invasions, and many other ancillary threats plaguing contemporary tropical fragmented landscapes^25^. The recovery here documented is therefore likely to represent a best-case scenario and patterns reported might be harder to observe under conditions that increasingly characterize the majority of human-modified tropical landscapes.

The sole reliance on mist-netting data precludes a complete overview of the effects of second forest regeneration on the BDFFP chiropteran fauna as a whole since, with the exception of *P*. *parnellii*, Amazonian aerial insectivorous bats (a diverse group that includes the families Thyropteridae, Furipteridae, Mormoopidae, Emballonuridae, Vespertilionidae, Molossidae and Natalidae)^32^ are not effectively sampled with mist-nets^33^. However, we anticipate that old-growth specialist aerial insectivores are likely to have benefited from the maturation of second growth surrounding the BDFFP fragments in a similar way than their phyllostomid counterparts and to the aerial insectivore *P*. *parnellii*.

Our results contrast with the catastrophic faunal declines observed in rodent communities by Gibson *et al*.^11^ in the forest islands of the Chiew Larn reservoir in Thailand. Whereas most species of the mega-diverse bat assemblage at the BDFFP increased in occupancy and abundance across the second growth-dominated landscape, in the land-bridge island system in Thailand and, during a similar time window (~20 years, Gibson *et al*.^11^; ~15 years, our study), most species became extinct in forest fragments surrounded by a static matrix (water). The recovery observed at the BDFFP was mostly due to the recolonization of previously deforested areas and forest fragments by specialist species, which increased in all sampled habitats during the second period. This recolonization is likely attributable to an increased diversity of food resources in the matrix, allowing to fulfil the energetic requirements of a larger set of species other than generalists. Additionally, many specialist bats at the BDFFP are highly edge-sensitive^34–36^ and consequently the maturation of the secondary vegetation adjoining fragment edges might have increased habitat suitability by reducing the magnitude of edge effects across the landscape. Notwithstanding major morphological and ecological differences between rodents and bats, the widely different trajectories exhibited by assemblages inhabiting true island systems^11^ and fragments embedded within a regenerating matrix (this study) highlight the potential of second growth forests to mitigate fragmentation-related extinctions.

In spite of the signs of recovery exhibited by specialist species across our study landscape ~30 years after forest clearance, our results do not support an increase in assemblage similarity between continuous forest and secondary forest over time. This, together with evidence that bat assemblages in smaller fragments (≤10 ha) and secondary forest sites still differ considerably from continuous forest in terms of species richness, evenness, composition and abundance^34,37^, suggests that the second growth matrix at the BDFFP still acts as an environmental filter. This filtering shapes bat assemblages in a trait-mediated manner, selectively benefiting bat species with a phytophagous diet and reduced body mass^38,39^. Similar pervasive consequences of forest clearance can still be detected in birds^40–42^ and primates^43^ in the BDFFP landscape, highlighting that, although second growth can be of conservation significance, primary forest is of irreplaceable value^23,44^.

Our results have important implications for the interpretation of land-use change studies using space-for-time approaches. Researchers rarely have the opportunity to collect data prior to the main disturbance events that mould humanized landscapes. Consequently, studies often have to rely on nearby sites where the target impact has not yet taken place and assume that these accurately mimic pre-disturbance conditions^45^. Here, we show that the species richness of generalists and both the species richness and abundance of specialists have increased in our reference sites in continuous forest, indicating considerable temporal heterogeneity in undisturbed forest assemblages over a period of ~15 years. This suggests that space-for-time results may be undermined not only by confounding effects arising from spatial heterogeneity but also by constraints associated with the temporal heterogeneity of the assemblages inhabiting sites used as spatial surrogates. This shifting baseline somewhat limits our capacity to attribute the observed changes in fragment and secondary forest bat assemblages entirely to the effect of matrix maturation. However, the contrasting temporal trends in the species richness of generalists in continuous forest and secondary forest, i.e. increase in continuous forest vs. decrease in secondary forest, indicate that secondary forest regeneration plays an important role in the assemblage dynamics across the landscape. Yet, our limited knowledge of the extent of spatial and temporal dynamics of generalist and specialist species in continuous forest and how these fluctuations may relate to patterns in fragmented landscapes still precludes a full understanding of these systems and therefore should be a high priority for future research.

Despite the controlled, experimental conditions of the BDFFP, our findings add to an increasing body of evidence (e.g.^9,15^) emphasizing that the transposition of patterns of biodiversity persistence in island ecosystems to fragmented terrestrial settings can be hampered by the dynamic nature of human-dominated landscapes, and consequently predictions under the island biogeographic framework can distort our understanding and misguide conservation strategies. Accordingly, and in light of the contrasting temporal trajectories of specialist and generalist species at the BDFFP, alternative theoretical frameworks, importantly, countryside biogeography^46^, in which species’ differential habitat affinities can be accommodated, emerge as better suited for forecasting biological changes in human-modified landscapes^9^.

In spite of some noteworthy regional declines in deforestation rates (e.g. Brazilian rainforests), tropical forest loss has increased by more than 2,000 km^2^/year since the beginning of the millennium^47^. Much of these deforested areas will be used to meet the growing demands for food and biofuel of an increasing human population^48^. However, following forest clearing, some converted areas are allowed to regenerate, giving rise to human-modified landscapes in which secondary forests account for an increasing proportion of total forest cover^16^. Our results, although contingent on the existence of nearby source populations, add to the evidence that secondary forests offer a tremendous opportunity for both assisted and non-assisted habitat restoration^49^. Among bats, frugivorous species are effective seed dispersers, especially of pioneer plant species^50^ and gleaning insectivores play essential roles in the reduction of herbivory levels through trophic control of herbivorous arthropods^51^. Populations able to persist in primary forest remnants can therefore enhance second growth successional processes and by doing so, aid in maintaining the provision of ecosystem services and improve habitat quality and connectivity in regenerating tropical forests.

To a large extent, the conservation potential of the world’s tropical secondary rainforests depends on the legal framework underpinning their governance. In the Brazilian Amazon, the state of Pará has recently introduced legislation recommending protection of >20-year-old secondary forest (as identified through inspection of satellite images) as well as younger stands depending on the total stand basal area of native trees and palms^52^. Although legal protection per se does not ensure long-term safeguarding of the services provided by second-growth forests, it represents a critical step towards their management. We therefore urge researchers, practitioners and policy makers to adopt similar protective measures, especially in areas where primary forest is scarce or highly fragmented.

Human-modified tropical landscapes are in continuous flux, with areas of secondary forest being converted to agricultural land and vice-versa. Vegetation disturbances, both anthropogenic and natural (e.g. fire), are irregular in space and time, moulding mosaic landscapes in which the classic split between fragments and matrix is blurred^53^. The ability of species to persist in such dynamic landscapes will ultimately depend on the interaction between their intrinsic traits (e.g. mobility and life span), interspecific interactions and the availability of habitat capable of meeting their specific resource needs. Although hotly debated^23,54,55^, the “rescue” potential of secondary forests in these dynamic landscapes is far from negligible^16^. While adding to mounting evidence that secondary forests are of conservation value, our, and many other long-term studies at the BDFFP (reviewed by Laurance *et al*.)^25^ and elsewhere in the tropics (e.g.)^17,56^ reveal that continuous primary forest and large (>100 ha) forest fragments are of overwhelming importance for the conservation of tropical biodiversity.

Our results show that specialist bats, which occurred at low abundances in secondary regrowth and in forest fragments ~15 years after the experimental clearing, have benefited from the increased permeability of the matrix associated with the maturation of the secondary forest in the matrix during the last 15 years. This suggests that matrix management, and specifically the management of regenerating (secondary) forest can majorly dictate the future of biodiversity in human-modified landscapes, including that remaining in fragments of natural vegetation.

## Material and Methods

### Study area

Bat surveys took place at the Biological Dynamics of Forest Fragments Project (BDFFP), approximately 80 km north of Manaus (2°30′S, 60°W, 30–125 m above sea level), state of Amazonas, Brazil (Fig. 1). Forest in the ~1,000 km^2^ study area is non-flooded (*terra firme*) rainforest with a canopy height of ca. 23 m and emergent trees reaching 55 m^57^. The forest at the BDFFP is among the most biodiverse in the world (tree species richness often exceeding 280 species/ha)^58^ and, with the exception of the experimental fragmentation, has been sheltered from anthropogenic disturbances such as logging and fires. The climate is characterized by a dry season between June and October and annual rainfall varies from 1,900 to 3,500 mm. Eleven fragments were isolated from continuous forest by distances of 80–650 m in the early 1980s and are categorized into size classes of 1, 10 and 100 ha. Fragments were originally located within cattle ranches (3,000–5,000 ha each) but poor soils and low productivity dictated the abandonment of livestock activities and fragments became gradually surrounded by secondary forest dominated mainly by *Vismia* spp. and *Cecropia* spp.^25^. Following secondary forest proliferation, fragment isolation was maintained by clearing a 100 m-wide strip of regrowth at intervals of ~10 years around most experimental forest fragments^35^. During this study fragment re-isolation occurred between 1999 and 2001. For a description of the study landscape experimental manipulation and ecosystem-wide responses see Laurance *et al*.^25^.

### Bat sampling

In both study periods (1996–2002 and 2011–2013) we sampled bats in forest fragments (six sites, three of 1 ha and three of 10 ha), secondary forest (seven sites) and continuous forest (six sites) (Fig. 1). Sampling started at dusk and nets were deployed until 0:00 am, being revised at intervals of ~20 minutes. Bias in capture rates due to net shyness was avoided by spacing visits to the same site by periods of three to four weeks and sampling was interrupted during heavy rains.

During the first sampling period bats were surveyed from January 1996 to June 1999 in forest fragments and continuous forest sites^59^, and from October 2001 to November 2002 in secondary forest^60^. The mist-netting protocol consisted of eight (secondary forest sites) and 18 to 24 (fragments and continuous forest sites) ground-level mist nets (12 m × 2.5 m) placed along existing trails. Trails used for sampling forest fragments were located as close as possible to the centre of the fragment. We surveyed fragment and continuous forest sites on seven to 12 nights and secondary forest sites between three to seven nights. Total mist net effort was 8,757, 9,429 and 860 mist-net hours (mnh; 1 mnh equals one 12 m net open for 1 h) for continuous forest, fragments and secondary forests, respectively. Captured bats were identified to species-level and had standard morphometric and demographic data collected. For this first study period, detailed site descriptions, methods and results for fragments and continuous forest can be found in Sampaio^61^ and Sampaio *et al*.^59^ and for secondary forest in Bobrowiec & Gribel^60^. Our analyses are restricted to ground-level captures in fragment and continuous forest interiors^59^ and to captures in *Vismia*- and *Cecropia*-dominated secondary forest^60^. Distance between sampling sites ranged from 148 m to 41 km and consequently some level of non-independence between bat assemblages of sites located closer together is plausible.

During the second period we re-surveyed all 19 sites between August 2011 and June 2013. The mist-netting protocol consisted of seven (secondary forest sites) and 14 (fragments and continuous forest sites) ground-level mist nets (12 × 2.5 m) placed at existing trails. Total mist net effort was 4,009, 3,963 and 1,941 mnh for continuous forest, fragments and secondary forests, respectively. Similarly to the first period, captured bats were identified and had standard morphometric and demographic data collected.

Bat capture and handling was conducted following guidelines approved by the American Society of Mammalogists^62^ and in accordance with Brazilian conservation and animal welfare laws. Sampling guidelines were approved by the ICMBio (Instituto Chico Mendes de Conservação da Biodiversidade) and research was conducted under permit number 26877–2.

We restricted our analyses to phyllostomid bats and *Pteronotus parnellii* since all other captured species are inadequately sampled with ground-level mist-nets^33^. Taxonomy follows Gardner^63^.

### Species affinities to primary and secondary forest

We used the statistical approach developed by Chazdon *et al*.^64^ to classify species into one of four groups: primary forest specialists, secondary forest specialists, generalists or too rare to classify. Classification was based on the whole dataset of 10,311 captures of 50 species sampled at the BDFFP between 1996 and 2014. Only a sub-set of these captures (6,109) was subsequently used in the joint species distribution models (see below). The method uses a multinominal model based on species relative abundance in both habitats (here defined as continuous primary forest vs forest fragments and secondary forest) and simultaneously minimizes bias due to different sampling effort between habitats and due to insufficient captures of rare species. Classification was conducted in R v.3.0.2^65^ using function *clamtest* of the *vegan* package and was based on the super-majority specialization threshold (K = 2/3) and setting a significance level of P = 0.01. We conservatively grouped primary forest specialists and species too rare to classify into a single group and since only two species were assigned to the secondary forest category, they were lumped together with generalists. We therefore considered two functional groups in our analysis: primary forest species and species too rare to classify (hereafter “specialist species”) and generalists and secondary forest specialists (hereafter “generalist species”).

### Joint species distribution model

We applied a joint species distribution model^27^ to relate the bat occurrence data to environmental covariates. As a sampling unit, we considered one mist-netting session in one site (n = 301 mist-netting sessions) (the study design is illustrated in Supplementary Fig. S2). As the data involved a large fraction of zeros (70%), we applied a hurdle model, thus modelling separately presence-absence (model 1), and abundance conditional on presence (model 2). In model 1, the response variable was the vector of presence-absences of all the 50 species, and we assumed a Bernoulli distribution with a probit link-function (**Y** matrix, Supplementary Fig. S2). In model 2, the response variable was the vector of abundances of those species which were present, whereas species that were absent were considered as missing data (**Y** matrix, Supplementary Fig. S2). In this case, we assumed an overdispersed Poisson distribution with a log-link function. Abundance was measured as the number of captured individuals, of which we subtracted one to match the range of the assumed distribution (overdispersed Poisson) with the range of the response variable (note that conditional on presence, the smallest value for number of individuals is one, not zero). As explanatory variables, we included habitat type (categorical: continuous forest, fragment, or secondary forest), survey period (first (1996–2002) or second survey (2011–13)), percentage of secondary forest cover within a radius of 500 m from each site and the log-transformed survey effort, measured as mist-net hours (**X** matrix, Supplementary Fig. S2). We also included an interaction between survey period and habitat type, as well as an interaction between survey period and secondary forest cover. Percent secondary forest cover was measured from a detailed digital map of the BDFFP landscape based on Landsat Thematic Mapper data from 1996 (for the first survey period) and 2011 (for the second survey period) – see Carreiras *et al*.^66^ for image classification details. A buffer size of 500 m was selected so as to minimize overlap between neighbouring sites.

To account for repeated measurements at the same sites, we assumed a site-level random effect, implemented at the community level using the latent factor approach of Ovaskainen *et al*.^67^. As species traits, we included the classification into habitat generalists and specialists (**T** matrix, Supplementary Fig. S2). To account for phylogenetic non-independence, we followed Abrego *et al*.^68^ to structure the error variance with a phylogenetic correlation matrix, derived from a phylogenetic tree under the diffusion model (**C** matrix, Supplementary Fig. S2). The phylogenetic tree was taken from Jones *et al*.^69^. We fitted the model in the Bayesian framework using the Gibbs sampler of Ovaskainen *et al*.^66,70^. We used the hierarchical modelling of species communities (HMSC) software for MatLab (HMSC-MatLab) to fit the model to the data, assuming the default priors described in the Supporting Information of Ovaskainen *et al*.^27^. We ran the model for 50,000 iterations out of which 15,000 were discarded as transient.

We used the parameterized model to predict the expected species richness and number of captured individuals (for all species and separately for generalists and specialists) in each habitat class and study period per survey visit. Species richness was computed as the sum (over the species) of the occurrence probabilities predicted by model 1. Number of individuals was computed as the sum (over the species) of species-specific abundances, computed as the product of occurrence probability (from model 1) and abundance conditional on presence (prediction of model 2 plus one). In these predictions, we standardized the mist netting effort to the mean value of a given habitat category across both study periods, and the percentage of secondary forest to the mean value of a particular habitat type during a given survey period. Capture effort was standardized within each habitat category and thus the results are comparable between periods but not across habitat types. Species-level responses were assessed by computing the difference between the occurrence probability and mean number of individuals expected to be captured per survey visit between the first and the second period.

Turnover metrics are considered better suited to quantify biodiversity change in local assemblages through time than simple temporal trends of within-sample diversity (temporal α diversity)^71^. To characterize assemblage turnover, we computed assemblage similarity between the different habitat categories as well as between the two study periods. Assemblage similarity was defined as the correlation between model-predicted occurrence probabilities or abundances (both log-transformed)^72^ We performed these calculations for all species, and separately for generalists and specialists only.

The data used in this study are archived at 10.6084/m9.figshare.5907322.v2.

## Electronic supplementary material

Supplementary Information
