# Supplementary material for: Secondary forest regeneration benefits old-growth specialist bats in a fragmented tropical landscape
Source: Sci Rep. 2018 Feb 28;8:3819. doi: 10.1038/s41598-018-21999-2 (PMC5830632; doi:10.1038/s41598-018-21999-2)
Supplement: Supplementary file 1 — Supplementary Information [file 41598_2018_21999_MOESM1_ESM.doc]

**Supplementary Material**

**Secondary forest regeneration benefits old-growth specialist bats in a fragmented tropical landscape**

Ricardo Rocha, Otso Ovaskainen, Adrià López-Baucells, Fábio Z. Farneda, Erica M. Sampaio, Paulo E.D. Bobrowiec, Mar Cabeza, Jorge M. Palmeirim and Christoph F.J. Meyer

***^*^ Corresponding author:*** *Ricardo Rocha;* ***E-mail:*** *ricardo.nature@gmail.com*

**This supplementary material contains:**

**Figure S1.** Bat species richness and abundance in continuous forest, fragments and secondary forest sites, ~15 years and ~30 years after experimental forest clearance.

**Figure S2.** Data used for the joint species distribution model.

**Table S1.** Bat species sampled at the BDFFP, Central Amazon, Brazil, ~15 years and ~30 years after forest clearing.

**Table S2.** Species-specific predictions for occupancy probability and abundance, ~15 years and ~30 years after experimental forest clearance.

**Table S3.** Assemblage similarity between continuous forest and modified habitats (fragments and secondary forest), ~15 years and ~30 years after experimental forest clearance.


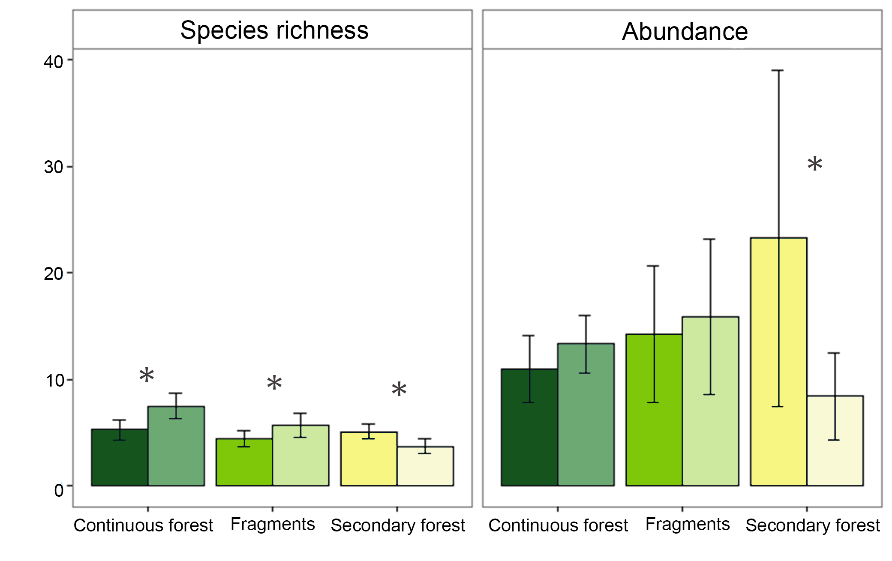


**Fig. S1.** **Bat species richness and abundance in continuous forest, fragments and secondary forest, ~15 years (dark colours) and ~30 years (light colours) after experimental forest clearance.** Plotted are the predictions of the mean number of species and the mean number of captured individuals (± posterior standard deviation) of all species combined per survey visit. Capture effort was standardized within each habitat category and thus the results are comparable only between periods but not across habitat types. *Asterisks* stand for high statistical support (posterior probability > 95%) for the predictions of the posterior probabilities being higher or lower ~30 years after experimental forest clearance than in 1996-2002. Species’ habitat specificities are reported in Table S1 (for classification description see Methods).

**
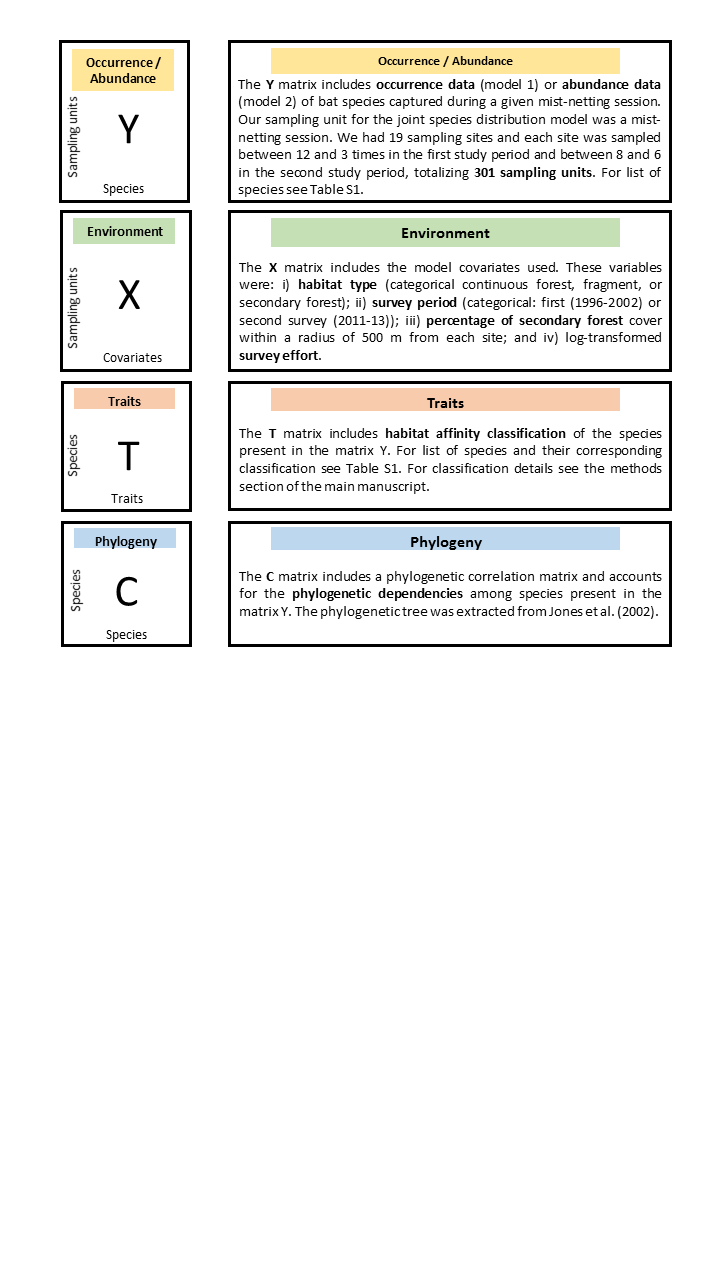
**

**Fig. S2.** **Explanation of data used for the joint species distribution model**. This figure is analogous to Figure 3 of Ovaskainen et al., 2017.

**Table S1.** **Bat species sampled at the Biological Dynamics of Forest Fragments Project, Central Amazon, Brazil, ~15 years (1996-2002) and ~30 years (2011-2013) after forest clearing.** Habitat abbreviations: CF = Continuous forest; F = Fragments; SF = Secondary forest.

| **Species** | **Habitat affinity** | **1996-2002** | | | **2011-2013** | | | **Total** |
| --- | --- | --- | --- | --- | --- | --- | --- | --- |
|  |  | **CF** | **F** | **SF** | **CF** | **F** | **SF** |  |
| *Artibeus cinereus* | Generalist | 11 | 24 | 2 | 8 | 6 | 4 | 55 |
| *Artibeus concolor* | Generalist | 12 | 17 | 22 | 3 | 4 | 18 | 76 |
| *Artibeus gnomus* | Generalist | 2 | 3 | 2 | 8 | 2 | 6 | 23 |
| *Artibeus obscurus* | Generalist | 70 | 148 | 10 | 14 | 18 | 7 | 267 |
| *Artibeus planirostris* | Generalist | 31 | 51 | 1 | 7 | 5 | 3 | 98 |
| *Carollia brevicauda* | Generalist | 59 | 57 | 31 | 13 | 25 | 31 | 216 |
| *Carollia perspicillata* | Generalist | 607 | 1076 | 618 | 181 | 530 | 353 | 3365 |
| *Desmodus rotundus* | Generalist | 14 | 18 | 1 | 7 | 1 | 1 | 42 |
| *Glossophaga soricina* | Generalist | 3 |  | 4 | 2 | 2 |  | 11 |
| *Lonchophylla thomasi* | Generalist | 15 | 20 |  | 7 | 15 | 3 | 60 |
| *Lophostoma schulzi* | Generalist | 11 |  |  | 2 | 1 | 1 | 15 |
| *Micronycteris megalotis* | Generalist | 8 | 7 | 3 | 1 | 1 | 1 | 21 |
| *Micronycteris microtis* | Generalist |  |  |  | 4 | 2 | 3 | 9 |
| *Mimon crenulatum* | Generalist | 29 | 12 | 2 | 15 | 9 | 5 | 72 |
| *Phylloderma stenops* | Generalist | 13 | 10 | 1 | 6 | 3 | 2 | 35 |
| *Phyllostomus discolor* | Generalist | 4 | 7 |  | 2 | 2 |  | 15 |
| *Rhinophylla pumilio* | Generalist | 160 | 211 | 81 | 74 | 131 | 83 | 740 |
| *Sturnira lilium* | Generalist |  | 14 | 2 |  |  | 1 | 17 |
| *Sturnira tildae* | Generalist | 3 | 21 | 12 | 1 | 1 | 9 | 47 |
| *Tonatia saurophila* | Generalist | 16 | 12 | 5 | 26 | 12 | 5 | 76 |
| *Trinycteris nicefori* | Generalist | 6 | 1 |  | 3 | 2 | 4 | 16 |
| *Uroderma bilobatum* | Generalist |  | 8 |  |  | 1 | 2 | 11 |
| *Vampyriscus bidens* | Generalist | 9 | 1 |  | 5 | 4 | 5 | 24 |
|  |  |  |  |  |  |  |  |  |
| *Ametrida centurio* | Specialist | 1 |  |  |  |  | 2 | 3 |
| *Anoura caudifer* | Specialist |  |  |  | 1 |  |  | 1 |
| *Artibeus lituratus* | Specialist | 39 | 23 | 4 | 16 | 2 | 8 | 92 |
| *Carollia castanea* | Specialist |  |  |  |  | 3 | 2 | 5 |
| *Chiroderma trinitatum* | Specialist | 5 | 1 |  |  |  |  | 6 |
| *Chiroderma villosum* | Specialist |  |  |  |  |  |  | 0 |
| *Choeroniscus minor* | Specialist | 2 | 3 |  | 1 | 5 |  | 11 |
| *Chrotopterus auritus* | Specialist | 1 | 2 |  | 2 | 1 | 1 | 7 |
| *Glyphonycteris daviesi* | Specialist |  |  |  | 1 |  | 2 | 3 |
| *Glyphonycteris sylvestris* | Specialist | 5 | 2 |  | 1 |  |  | 8 |
| *Lampronycteris brachyotis* | Specialist |  |  |  |  | 1 | 1 | 2 |
| *Lichonycteris degener* | Specialist | 1 |  |  |  |  |  | 1 |
| *Lophostoma brasiliense* | Specialist |  |  |  |  |  | 1 | 1 |
| *Lophostoma carrikeri* | Specialist |  |  |  |  | 1 | 1 | 2 |
| *Lophostoma silvicolum* | Specialist | 57 | 44 | 2 | 40 | 11 | 12 | 166 |
| *Mesophylla macconnelli* | Specialist | 20 | 4 | 1 | 10 | 2 | 2 | 39 |
| *Micronycteris hirsuta* | Specialist | 2 | 1 |  |  |  |  | 3 |
| *Micronycteris sanborni* | Specialist |  |  |  |  |  |  | 0 |
| *Micronycteris schmidtorum* | Specialist |  |  |  |  |  |  | 0 |
| *Phyllostomus elongatus* | Specialist | 17 | 3 | 5 | 14 | 5 | 3 | 47 |
| *Phyllostomus hastatus* | Specialist | 3 | 1 |  | 1 |  |  | 5 |
| *Platyrrhinus helleri* | Specialist |  | 1 |  |  |  | 1 | 2 |
| *Pteronotus parnellii* | Specialist | 68 | 27 | 5 | 74 | 24 | 29 | 227 |
| *Trachops cirrhosus* | Specialist | 68 | 10 | 1 | 52 | 26 | 7 | 164 |
| *Vampyressa pusilla* | Specialist |  |  |  |  |  |  | 0 |
| *Vampyriscus brocki* | Specialist |  |  |  |  | 2 |  | 2 |
| *Vampyrum spectrum* | Specialist | 1 |  |  |  |  |  | 1 |
| **Total** |  | **1373** | **1840** | **815** | **602** | **860** | **619** | **6109** |

**Table S2. Species-specific predictions for occupancy probability and abundance in continuous forest, fragments and secondary forest, ~15 years and ~30 years after experimental forest clearance.** Predictions account for within-habitat differences in capture effort between the two periods. Posterior probability stands for the probability of the predictions for occupancy and abundance being higher in 1996-2002 than in 2011-2013; values showing high statistical support (posterior probability > 95%) are highlighted in bold.

|  | **Occupancy** | | | **Abundance** | | |
| --- | --- | --- | --- | --- | --- | --- |
| **Species** | **1996-2002** | **2011-2013** | **Posterior probability** | **1996-2002** | **2011-2013** | **Posterior probability** |
| **Continuous Forest** |  |  |  |  |  |  |
| *Ametrida centurio* | 0.007 | 0.008 | 0.423 | 0.008 | 0.009 | 0.426 |
| *Anoura caudifer* | 0.004 | 0.015 | 0.814 | 0.005 | 0.015 | 0.814 |
| *Artibeus cinereus* | 0.080 | 0.191 | **0.966** | 0.099 | 0.209 | 0.946 |
| *Artibeus concolor* | 0.099 | 0.119 | 0.594 | 0.138 | 0.130 | 0.434 |
| *Artibeus gnomus* | 0.034 | 0.154 | **0.994** | 0.038 | 0.164 | **0.989** |
| *Artibeus lituratus* | 0.197 | 0.226 | 0.634 | 0.428 | 0.381 | 0.377 |
| *Artibeus obscurus* | 0.311 | 0.348 | 0.651 | 0.487 | 0.409 | 0.283 |
| *Artibeus planirostris* | 0.193 | 0.193 | 0.497 | 0.251 | 0.221 | 0.403 |
| *Carollia brevicauda* | 0.427 | 0.345 | 0.206 | 0.645 | 0.428 | 0.066 |
| *Carollia castanea* | 0.006 | 0.006 | 0.406 | 0.007 | 0.007 | 0.400 |
| *Carollia perspicillata* | 0.814 | 0.897 | 0.849 | 4.014 | 2.806 | 0.114 |
| *Chiroderma trinitatum* | 0.019 | 0.014 | 0.291 | 0.021 | 0.015 | 0.300 |
| *Chiroderma villosum* | 0.002 | 0.002 | 0.383 | 0.002 | 0.002 | 0.377 |
| *Choeroniscus minor* | 0.023 | 0.044 | 0.709 | 0.023 | 0.045 | 0.720 |
| *Chrotopterus auritus* | 0.021 | 0.045 | 0.840 | 0.022 | 0.052 | 0.851 |
| *Desmodus rotundus* | 0.097 | 0.241 | **0.960** | 0.114 | 0.267 | **0.951** |
| *Glossophaga soricina* | 0.032 | 0.052 | 0.689 | 0.034 | 0.055 | 0.689 |
| *Glyphonycteris daviesi* | 0.006 | 0.019 | 0.811 | 0.006 | 0.020 | 0.811 |
| *Glyphonycteris sylvestris* | 0.031 | 0.046 | 0.623 | 0.032 | 0.047 | 0.623 |
| *Lampronycteris brachyotis* | 0.003 | 0.006 | 0.666 | 0.003 | 0.006 | 0.660 |
| *Lichonycteris degener* | 0.006 | 0.008 | 0.506 | 0.006 | 0.008 | 0.503 |
| *Lonchophylla thomasi* | 0.099 | 0.169 | 0.823 | 0.115 | 0.197 | 0.829 |
| *Lophostoma brasiliense* | 0.005 | 0.012 | 0.720 | 0.005 | 0.015 | 0.743 |
| *Lophostoma carrikeri* | 0.005 | 0.014 | 0.763 | 0.006 | 0.016 | 0.789 |
| *Lophostoma schulzi* | 0.057 | 0.094 | 0.791 | 0.064 | 0.107 | 0.797 |
| *Lophostoma silvicolum* | 0.300 | 0.522 | **0.980** | 0.414 | 0.927 | **0.997** |
| *Mesophylla macconnelli* | 0.116 | 0.140 | 0.617 | 0.142 | 0.166 | 0.589 |
| *Micronycteris hirsuta* | 0.012 | 0.012 | 0.414 | 0.013 | 0.012 | 0.406 |
| *Micronycteris megalotis* | 0.067 | 0.072 | 0.500 | 0.069 | 0.073 | 0.503 |
| *Micronycteris microtis* | 0.011 | 0.051 | **0.951** | 0.012 | 0.053 | 0.949 |
| *Micronycteris sanborni* | 0.001 | 0.003 | 0.526 | 0.001 | 0.003 | 0.517 |
| *Micronycteris schmidtorum* | 0.001 | 0.002 | 0.523 | 0.001 | 0.002 | 0.523 |
| *Mimon crenulatum* | 0.158 | 0.242 | 0.826 | 0.216 | 0.375 | 0.891 |
| *Phylloderma stenops* | 0.088 | 0.112 | 0.631 | 0.103 | 0.154 | 0.717 |
| *Phyllostomus discolor* | 0.021 | 0.064 | 0.929 | 0.025 | 0.072 | 0.914 |
| *Phyllostomus elongatus* | 0.116 | 0.264 | **0.980** | 0.135 | 0.296 | **0.966** |
| *Phyllostomus hastatus* | 0.026 | 0.044 | 0.723 | 0.027 | 0.046 | 0.723 |
| *Platyrrhinus helleri* | 0.003 | 0.004 | 0.483 | 0.003 | 0.005 | 0.477 |
| *Pteronotus parnellii* | 0.532 | 0.642 | 0.791 | 0.834 | 1.756 | **0.994** |
| *Rhinophylla pumilio* | 0.633 | 0.752 | 0.889 | 1.567 | 1.569 | 0.523 |
| *Sturnira lilium* | 0.005 | 0.006 | 0.437 | 0.006 | 0.006 | 0.434 |
| *Sturnira tildae* | 0.030 | 0.047 | 0.683 | 0.042 | 0.057 | 0.603 |
| *Tonatia saurophila* | 0.111 | 0.337 | **0.997** | 0.138 | 0.481 | **1.000** |
| *Trachops cirrhosus* | 0.360 | 0.672 | **1.000** | 0.522 | 1.382 | **1.000** |
| *Trinycteris nicefori* | 0.049 | 0.097 | 0.806 | 0.050 | 0.098 | 0.800 |
| *Uroderma bilobatum* | 0.005 | 0.008 | 0.586 | 0.006 | 0.009 | 0.551 |
| *Vampyressa pusilla* | 0.002 | 0.002 | 0.543 | 0.002 | 0.003 | 0.531 |
| *Vampyriscus bidens* | 0.004 | 0.008 | 0.677 | 0.004 | 0.008 | 0.674 |
| *Vampyriscus brocki* | 0.044 | 0.108 | 0.940 | 0.054 | 0.116 | 0.891 |
| *Vampyrum spectrum* | 0.006 | 0.011 | 0.626 | 0.007 | 0.012 | 0.629 |
| **Fragments** |  |  |  |  |  |  |
| *Ametrida centurio* | 0.004 | 0.010 | 0.689 | 0.004 | 0.011 | 0.686 |
| *Anoura caudifer* | 0.001 | 0.008 | 0.811 | 0.001 | 0.009 | 0.817 |
| *Artibeus cinereus* | 0.139 | 0.144 | 0.489 | 0.190 | 0.161 | 0.346 |
| *Artibeus concolor* | 0.139 | 0.119 | 0.374 | 0.201 | 0.138 | 0.237 |
| *Artibeus gnomus* | 0.024 | 0.067 | 0.883 | 0.029 | 0.071 | 0.866 |
| *Artibeus lituratus* | 0.134 | 0.059 | 0.089 | 0.237 | 0.086 | 0.069 |
| *Artibeus obscurus* | 0.328 | 0.261 | 0.243 | 0.676 | 0.343 | **0.029** |
| *Artibeus planirostris* | 0.191 | 0.150 | 0.323 | 0.311 | 0.173 | 0.134 |
| *Carollia brevicauda* | 0.377 | 0.383 | 0.517 | 0.632 | 0.728 | 0.677 |
| *Carollia castanea* | 0.005 | 0.045 | **0.951** | 0.006 | 0.068 | **0.969** |
| *Carollia perspicillata* | 0.928 | 0.955 | 0.700 | 8.250 | 7.816 | 0.377 |
| *Chiroderma trinitatum* | 0.008 | 0.009 | 0.466 | 0.009 | 0.009 | 0.460 |
| *Chiroderma villosum* | 0.001 | 0.002 | 0.597 | 0.001 | 0.002 | 0.597 |
| *Choeroniscus minor* | 0.024 | 0.097 | 0.940 | 0.025 | 0.103 | 0.943 |
| *Chrotopterus auritus* | 0.009 | 0.026 | 0.800 | 0.010 | 0.030 | 0.823 |
| *Desmodus rotundus* | 0.099 | 0.077 | 0.357 | 0.123 | 0.087 | 0.300 |
| *Glossophaga soricina* | 0.009 | 0.048 | 0.931 | 0.010 | 0.052 | 0.937 |
| *Glyphonycteris daviesi* | 0.002 | 0.008 | 0.794 | 0.002 | 0.008 | 0.794 |
| *Glyphonycteris sylvestris* | 0.013 | 0.012 | 0.400 | 0.013 | 0.013 | 0.417 |
| *Lampronycteris brachyotis* | 0.001 | 0.016 | **0.963** | 0.001 | 0.017 | **0.966** |
| *Lichonycteris degener* | 0.003 | 0.010 | 0.743 | 0.003 | 0.012 | 0.757 |
| *Lonchophylla thomasi* | 0.149 | 0.223 | 0.809 | 0.170 | 0.353 | 0.920 |
| *Lophostoma brasiliense* | 0.001 | 0.008 | 0.903 | 0.001 | 0.009 | 0.911 |
| *Lophostoma carrikeri* | 0.002 | 0.018 | **0.969** | 0.002 | 0.020 | **0.974** |
| *Lophostoma schulzi* | 0.008 | 0.049 | 0.949 | 0.010 | 0.053 | 0.937 |
| *Lophostoma silvicolum* | 0.245 | 0.259 | 0.540 | 0.301 | 0.349 | 0.617 |
| *Mesophylla macconnelli* | 0.034 | 0.053 | 0.726 | 0.037 | 0.059 | 0.717 |
| *Micronycteris hirsuta* | 0.004 | 0.007 | 0.560 | 0.005 | 0.008 | 0.580 |
| *Micronycteris megalotis* | 0.054 | 0.053 | 0.480 | 0.055 | 0.055 | 0.477 |
| *Micronycteris microtis* | 0.006 | 0.049 | **0.969** | 0.006 | 0.053 | **0.971** |
| *Micronycteris sanborni* | 0.000 | 0.003 | 0.729 | 0.000 | 0.003 | 0.734 |
| *Micronycteris schmidtorum* | 0.000 | 0.002 | 0.734 | 0.000 | 0.002 | 0.740 |
| *Mimon crenulatum* | 0.051 | 0.154 | **0.986** | 0.069 | 0.201 | **0.977** |
| *Phylloderma stenops* | 0.070 | 0.088 | 0.606 | 0.077 | 0.114 | 0.657 |
| *Phyllostomus discolor* | 0.037 | 0.053 | 0.611 | 0.042 | 0.070 | 0.657 |
| *Phyllostomus elongatus* | 0.033 | 0.107 | **0.960** | 0.035 | 0.126 | **0.969** |
| *Phyllostomus hastatus* | 0.007 | 0.012 | 0.631 | 0.007 | 0.014 | 0.646 |
| *Platyrrhinus helleri* | 0.007 | 0.009 | 0.497 | 0.008 | 0.010 | 0.489 |
| *Pteronotus parnellii* | 0.202 | 0.316 | 0.840 | 0.266 | 0.558 | 0.931 |
| *Rhinophylla pumilio* | 0.706 | 0.836 | 0.926 | 1.886 | 2.567 | 0.926 |
| *Sturnira lilium* | 0.074 | 0.014 | **0.029** | 0.091 | 0.015 | **0.017** |
| *Sturnira tildae* | 0.107 | 0.050 | 0.109 | 0.182 | 0.063 | 0.054 |
| *Tonatia saurophila* | 0.078 | 0.207 | **0.994** | 0.092 | 0.265 | **0.994** |
| *Trachops cirrhosus* | 0.067 | 0.372 | **1.000** | 0.081 | 0.714 | **1.000** |
| *Trinycteris nicefori* | 0.011 | 0.064 | **0.963** | 0.011 | 0.066 | **0.963** |
| *Uroderma bilobatum* | 0.035 | 0.039 | 0.517 | 0.045 | 0.044 | 0.460 |
| *Vampyressa pusilla* | 0.001 | 0.003 | 0.794 | 0.001 | 0.003 | 0.794 |
| *Vampyriscus bidens* | 0.001 | 0.022 | **0.991** | 0.001 | 0.024 | **0.989** |
| *Vampyriscus brocki* | 0.014 | 0.091 | **0.980** | 0.017 | 0.107 | **0.977** |
| *Vampyrum spectrum* | 0.001 | 0.006 | 0.803 | 0.001 | 0.006 | 0.806 |
| **Secondary forest** |  |  |  |  |  |  |
| *Ametrida centurio* | 0.004 | 0.026 | 0.940 | 0.004 | 0.027 | 0.946 |
| *Anoura caudifer* | 0.002 | 0.002 | 0.671 | 0.002 | 0.002 | 0.677 |
| *Artibeus cinereus* | 0.114 | 0.058 | 0.166 | 0.131 | 0.068 | 0.189 |
| *Artibeus concolor* | 0.359 | 0.100 | **0.003** | 0.639 | 0.245 | **0.040** |
| *Artibeus gnomus* | 0.076 | 0.083 | 0.554 | 0.081 | 0.091 | 0.563 |
| *Artibeus lituratus* | 0.138 | 0.049 | 0.080 | 0.151 | 0.069 | 0.131 |
| *Artibeus obscurus* | 0.350 | 0.109 | **0.003** | 0.376 | 0.118 | **0.003** |
| *Artibeus planirostris* | 0.086 | 0.038 | 0.174 | 0.088 | 0.039 | 0.180 |
| *Carollia brevicauda* | 0.650 | 0.312 | **0.000** | 1.192 | 0.492 | **0.003** |
| *Carollia castanea* | 0.006 | 0.039 | 0.946 | 0.007 | 0.044 | 0.949 |
| *Carollia perspicillata* | 0.988 | 0.877 | **0.003** | 16.277 | 4.338 | **0.000** |
| *Chiroderma trinitatum* | 0.003 | 0.005 | 0.754 | 0.003 | 0.005 | 0.757 |
| *Chiroderma villosum* | 0.000 | 0.002 | 0.900 | 0.000 | 0.002 | 0.906 |
| *Choeroniscus minor* | 0.007 | 0.006 | 0.523 | 0.007 | 0.006 | 0.520 |
| *Chrotopterus auritus* | 0.004 | 0.014 | 0.883 | 0.004 | 0.014 | 0.886 |
| *Desmodus rotundus* | 0.054 | 0.018 | 0.200 | 0.056 | 0.018 | 0.197 |
| *Glossophaga soricina* | 0.138 | 0.012 | **0.000** | 0.139 | 0.012 | **0.000** |
| *Glyphonycteris daviesi* | 0.001 | 0.019 | **0.971** | 0.001 | 0.020 | **0.971** |
| *Glyphonycteris sylvestris* | 0.003 | 0.008 | 0.800 | 0.003 | 0.008 | 0.803 |
| *Lampronycteris brachyotis* | 0.001 | 0.013 | **0.989** | 0.001 | 0.013 | **0.989** |
| *Lichonycteris degener* | 0.001 | 0.002 | 0.814 | 0.001 | 0.002 | 0.814 |
| *Lonchophylla thomasi* | 0.029 | 0.028 | 0.583 | 0.033 | 0.032 | 0.597 |
| *Lophostoma brasiliense* | 0.001 | 0.009 | **0.969** | 0.001 | 0.010 | **0.969** |
| *Lophostoma carrikeri* | 0.001 | 0.012 | **0.989** | 0.001 | 0.012 | **0.989** |
| *Lophostoma schulzi* | 0.012 | 0.017 | 0.711 | 0.013 | 0.018 | 0.709 |
| *Lophostoma silvicolum* | 0.104 | 0.147 | 0.726 | 0.110 | 0.160 | 0.734 |
| *Mesophylla macconnelli* | 0.028 | 0.039 | 0.694 | 0.028 | 0.042 | 0.714 |
| *Micronycteris hirsuta* | 0.001 | 0.004 | 0.806 | 0.001 | 0.004 | 0.803 |
| *Micronycteris megalotis* | 0.091 | 0.023 | 0.066 | 0.095 | 0.023 | 0.054 |
| *Micronycteris microtis* | 0.011 | 0.037 | 0.903 | 0.012 | 0.039 | 0.900 |
| *Micronycteris sanborni* | 0.000 | 0.002 | 0.889 | 0.000 | 0.002 | 0.889 |
| *Micronycteris schmidtorum* | 0.000 | 0.001 | 0.931 | 0.000 | 0.001 | 0.931 |
| *Mimon crenulatum* | 0.084 | 0.061 | 0.331 | 0.095 | 0.069 | 0.343 |
| *Phylloderma stenops* | 0.045 | 0.022 | 0.251 | 0.050 | 0.025 | 0.257 |
| *Phyllostomus discolor* | 0.015 | 0.008 | 0.383 | 0.023 | 0.010 | 0.363 |
| *Phyllostomus elongatus* | 0.074 | 0.034 | 0.177 | 0.090 | 0.040 | 0.174 |
| *Phyllostomus hastatus* | 0.004 | 0.003 | 0.580 | 0.004 | 0.003 | 0.580 |
| *Platyrrhinus helleri* | 0.002 | 0.010 | 0.949 | 0.002 | 0.011 | **0.951** |
| *Pteronotus parnellii* | 0.169 | 0.351 | **0.954** | 0.193 | 0.473 | **0.974** |
| *Rhinophylla pumilio* | 0.896 | 0.692 | **0.014** | 2.621 | 1.285 | **0.000** |
| *Sturnira lilium* | 0.080 | 0.020 | 0.094 | 0.085 | 0.022 | 0.091 |
| *Sturnira tildae* | 0.245 | 0.069 | **0.014** | 0.349 | 0.100 | **0.020** |
| *Tonatia saurophila* | 0.134 | 0.091 | 0.263 | 0.151 | 0.101 | 0.254 |
| *Trachops cirrhosus* | 0.064 | 0.101 | 0.783 | 0.070 | 0.112 | 0.780 |
| *Trinycteris nicefori* | 0.014 | 0.057 | 0.929 | 0.015 | 0.058 | 0.920 |
| *Uroderma bilobatum* | 0.008 | 0.026 | 0.863 | 0.009 | 0.029 | 0.869 |
| *Vampyressa pusilla* | 0.000 | 0.003 | 0.946 | 0.000 | 0.003 | 0.949 |
| *Vampyriscus bidens* | 0.001 | 0.008 | 0.949 | 0.001 | 0.008 | 0.949 |
| *Vampyriscus brocki* | 0.021 | 0.058 | 0.880 | 0.022 | 0.066 | 0.889 |
| *Vampyrum spectrum* | 0.000 | 0.003 | 0.906 | 0.000 | 0.003 | 0.906 |

**Table S3.** **Assemblage similarity between continuous forest and modified habitats** **(fragments and secondary forest), ~15 years and ~30 years after experimental forest clearance.** Values correspond to the between-habitat correlation of the model’s predictions of occurrence probabilities and abundance (log-transformed), computed for those two environments. Posterior probabilities stand for the probability of the assemblage similarity between continuous forest and the modified habitat (fragments or secondary forest) being lower in first than in the second period.

|  | **Fragments** | | **Secondary Forest** | |
| --- | --- | --- | --- | --- |
|  | *Occupancy* | *Abundance* | *Occupancy* | *Abundance* |
| *1996-2002* | 0.833 | 0.846 | 0.788 | 0.8 |
| *2011-2013* | 0.795 | 0.807 | 0.67 | 0.678 |
| *Posterior probability* | 0.309 | 0.329 | 0.08 | 0.09 |
